# Retrospective Study to Reduce Blood Transfusion Waste in Remote Island Healthcare Settings

**DOI:** 10.1155/2023/5549655

**Published:** 2023-12-12

**Authors:** Takamitsu Sakamoto, Hiroyuki Mizuta, Naohisa Niiro, Teruyoshi Amagai

**Affiliations:** ^1^Fukuoka Tokushukai Medical Center, Department of General Medicine, Kasuga, Japan; ^2^The Graduate School of Medical Sciences, Kumamoto University, Kumamoto, Japan; ^3^Isen Clinic, Kagoshima, Japan; ^4^Tokunoshima Tokushukai Hospital, Kagoshima, Japan; ^5^Faculty of Health Care Sciences, Department of Clinical Engineering, Jikei University of Health Care Sciences, Tokyo, Japan

## Abstract

**Background:**

Tokunoshima is a remote island in the Amami Islands, 470 km southwest of the Kagoshima mainland. It has a population of 23,000 and consists of three towns: Tokunoshima, Isen, and Amagi. Three medical institutions on the island are responsible for blood transfusion medicine, but there is no blood stockpiling station on the island, and blood is stockpiled in each of the hospitals. Although Tokunoshima Tokushukai Hospital is responsible for 70% of transfusion medicine on Tokunoshima, it is difficult to maintain a sufficient amount of blood in stock considering disposal.

**Aim:**

To determine whether changing the distribution of blood types in a hospital's stockpile would reduce the transfusion disposal rate.

**Methods:**

This was a retrospective survey. By changing the in-house stock of blood products for transfusions delivered to our hospital over 10 years from January 2013 to December 2017 (preintervention) and from January 2018 to December 2022 (postintervention), we compared the cost-saving effects of these two intervention strategies on disposal rates and blood inventories, as well as the survival rates of case profiles requiring transfusion interventions in hospital-donated transfusion and ABO-incompatible transfusion between two periods. The hospital's stock of RBC had changes that storage of type (A, B, O, AB) RBC from (4, 4, 4, 2) units in the pre-interventon to (2, 2, 6, 0) units in the postintervention.

**Results:**

The annual blood product waste rate decreased from 23.4% in the preintervention period to 17.9% in the post-intervention period.

**Conclusion:**

By changing the blood products stockpiled for transfusion medicine in Tokunoshima, the transfusion disposal rate can be reduced.

## 1. Introduction

**Figure 1:**
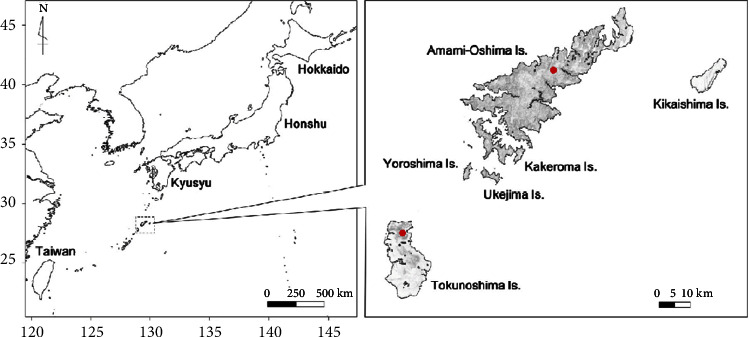
Map showing the location of the Amami Oshima and Tokunoshima Islands. The Tokunoshima Island is located 470 km south of the Kyushu Island, the true southern tip of Japan. It is an hour flight from Kagoshima City, the southernmost point of Kyushu.

Tokunoshima Island belongs to the Amami Islands, 470 km southwest of the Kagoshima mainland. It is a remote island with a population of 23,000 and a circumference of 90 km, consisting of three towns: Tokunoshima, Isen, and Amagi (Figure 1). Known as an island of longevity and fertility, the island has 45 oldest adults over 100 years old, as represented by Shigechiyo Izumi, and the total fertility rate of the three towns ranked first to third in Japan [1].

Tokunoshima Tokushukai Hospital (TTH) is a medium-sized hospital with 199 beds (120 acute care and 79 convalescent types), making it the largest in Tokunoshima, and it has also obtained Blood Transfusion Management Fee II certified to be reimbursed for every blood transfusion. The annual number of ambulances brought in was 887, and the number of surgeries was 118 in surgery, 164 in orthopedics, and 31 in gynecology between January and December 2020; several cases with critical bleeding due to trauma have been experienced. However, full-time surgeons and full-time orthopedic surgeons have been absent for years and are currently being maintained by regular support systems.

In Tokunoshima, three medical institutions provide blood transfusion services, but there are no blood storage facilities on the island, so each institution keeps its blood reserves at its own hospital. Although TTH is responsible for 70% of transfusion medicine in Tokunoshima, it is difficult to maintain sufficient quantities of blood in consideration of disposal; before the abolition of the blood storage station on the main island of Amami Oshima on April 1, 2018 [2], the irradiated red cell solution—LR (hereafter RBC) of type A, B, and O, 4 units each, and 2 units of type AB were permanently stocked in the hospital.

These blood products are transported by air from Kagoshima (Figure 1) and may be delayed due to hazardous weather conditions. The minimum time from ordering blood products to receiving them was 6 hours, and the maximum was 22 hours. In particular, under the COVID-19 pandemic environment since January 2020, the ability to bring in medical supplies from outside the island is extremely limited, and blood transfusion products are no exception.

Under these circumstances, it is extremely important to maintain a reserve of transfused blood, but at the same time, there is a trade-off between the amount of transfused blood storage and the economic burden because of the costs associated with disposal. To address these issues, in order to reduce the waste rate of blood products in remote islands with limited medical resources, we proposed the strategy of changing the amount of blood for transfusion as mentioned above from 2018 using inhospital-donated and ABO-incompatible transfusions; details are described in the methods session. This change was in line with a policy statement that blood for transfusion should not be collected at the hospital unless there are extraordinary circumstances in areas where the blood supply system from Japanese Red Cross blood centers has been established [3].

## 2. Aim

To determine whether the changes in our transfusion program reduce the waste rate of intended transfusion products and have a real economic impact, we measured every five consecutive years from 2013 to 2017, before the transfusion program was initiated. We examined the impact of the intervention between preintervention and postintervention on the waste rate and the monetary impact of transfusion.

## 3. Methods

This study was approved by the hospital's ethics committee, and an opt-out document was made publicly available. The approval number is 2021-02. This was a retrospective survey.

**Figure 2:**
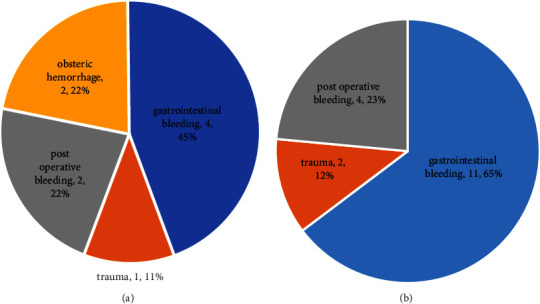
Case profiles of inhospital-donated transfusion and ABO-incompatible transfusion (the currency exchange rate was 140 yen to one US dollar). Case profiles with inhospital-donated transfusion (a) and ABO-incompatible transfusion (b). Gastrointestinal disease was the most common indication for both inhospital-donated and ABO-incompatible transfusions.

The 10-year study period from January 2013 to December 2017 and from January 2018 to December 2022 was divided into two periods: preintervention and postintervention, respectively. The intervention consisted of the following two: 1, in-house stock of blood products for transfusions (Figure 1); 2, inhospital donor transfusion (IDT) and ABO-incompatible transfusion (AIT), which are approved for use in emergencies according to guidelines [4], were left to the discretion of the attending physician whether or not to perform these two emergency transfusion methods, IDT and AIT, during preintervention, but IDT and/or AIT should be performed during postintervention in an emergency (Table 1).

We compared the cost-saving effects of these two intervention strategies on disposal rates and blood inventories, as well as the survival rates of case profiles requiring transfusion interventions IDT and AIT before and after intervention. Here, the disposal rate was defined as the disposal blood unit divided by the total stock transfusion unit. All study transfusions were indicated according to guidelines [4].

The survey items included age, gender, disease name, treatment, transfusion volume, outcome, and the Shock Index (SI) calculated by dividing the heart rate by the systolic blood pressure. The outcome was defined as death or rescue. This SI value is also used to classify the severity of hemorrhagic shock into four classes as follows: SI < 0.6, 0.6–1, 1–1.4, and ≥1.4 were defined as no shock (class I), mild shock (class II), moderate shock (class III), and severe shock (class IV), respectively.

## 4. Results

During this period, there were 18 cases of ABO-incompatible transfusions and nine cases of inhospital-donated transfusions.

### 4.1. Effects of the Disposal Rate and Economy

The annual blood product waste rate decreased from 23.4% in the preintervention period to 17.9% in the postintervention period (Tables 2 and 3). This reduced waste rate also reduced annual blood product disposal costs from US dollar (USD) 12,344 to 7,777, resulting in an annual savings of approximately USD 450.

Comparing the profile of ABO-incompatible transfusion cases before and after the intervention, there was no difference in the number of cases before and after the intervention (6 cases) and in the mortality rate (50%) (Table 4).

### 4.2. Survival Rates of Case Profiles Requiring Transfusion Interventions IDT and AIT

Looking at the profile of cases of donor transfusion in the hospital, comparing the cases before and after the intervention, the number decreased from 4 to 1. Among them, one died in the preperiod, the SI was class III, the transfusion volume was the largest with 10 units, the primary disease was a case of esophageal variceal rupture, and the background was thrombocytopenia and cirrhosis with coagulation factor deficiency (Table 5).

Of the 26 cases of ABO-incompatible and inhospital-donated transfusions, 15 were due to gastrointestinal bleeding (Figure 2) and the remainder were posttraumatic in three cases and surgical in six cases. Of the twenty-two cases (four duplicates, Table 6) in which ABO-incompatible and donated transfusions have been performed in the hospital, nine resulted in death, for a mortality rate of 40%.

## 5. Discussion

### 5.1. Withdrawal of Blood Storage Station Increases the Blood Disposal Rate in Remote Island Areas

Our hospital, located on a remote island in Kagoshima Prefecture, occasionally encounters situations where emergency blood transfers from reginal, the Kagoshima Red Cross Blood Center cannot be completed in time due to geographical circumstances. In addition, the blood storage station on Amami Oshima Island disappeared in April 2018, so to be prepared for emergencies, we examined the impact of the intervention between preintervention and postintervention on the wastage and monetary impact of transfusion. At first, our hospital decided to secure a certain amount of blood for transfusion in the hospital to discard it, but the annual amount of blood discarded amounted to 14200 USD, a situation that could put pressure on management considering the burden on the hospital as a private hospital. Ohki et al. reported that the withdrawal of the blood storage station from Amami Oshima resulted in an increase in the blood disposal rate at the core hospital in Amami Oshima and uneven distribution of medical resources [5]. The waste of valuable medical resources through blood donation was also seen as an ethical issue.

### 5.2. Designed and Implemented Measures to Maintain a Stable Blood Transfusion System at a Remote Island Healthcare Facility

Given the high rate of blood product waste, we decided to reduce the hospital's blood transfusion inventory by allowing ABO-incompatible transfusions. As a result, we designed two strategies to increase as follows: education of disposal transfusion after applying the intervention of the two systems, “inhospital-donated transfusion system” and “ABO-incompatible transfusion” of type O blood regardless of the donor's ABO blood group, and an increase in ABO-incompatible transfusion. As a result, contrary to our expectations, despite the taste of the redesigned new systems, no significant change has been observed. In the FY 2009 Blood Products Usage Survey Report, the national average disposal rate at facilities that obtained Transfusion Management Fee II and had no clinical transfusion nurse assigned to the facility was reported at 3.516% [6]. As a result of the Amami Blood Rotation (BR), the waste rate of type O red blood cells was dramatically reduced from 31.3% to 3.7% at Kagoshima Prefectural Oshima Hospital [7]. In the future, it will be necessary to develop medical measures to further reduce the waste rate, such as avoiding excessive blood collection by accurately estimating the amount of blood needed for transfusion.

### 5.3. Interpreting the Severity of Hemorrhagic Shock and Analyzing Results for Cases Requiring Emergency Blood Transfusion

In our island, transfusion cases were thought to be mainly caused by trauma, but contrary to expectations, the actual main cause of emergency transfusion was gastrointestinal bleeding.

In general, the survival rate of class II or higher cases in the SI classification is lower than that of class I cases. However, if we limit our analysis to class II cases, the mortality rate of cases with ABO-incompatible and inhospital-donated transfusions was 87.5% and 20%, respectively. Overall, eight patients were spared by ABO-incompatible transfusion and six patients by inhospital-donated blood transfusion. According to a report by Kiyotake et al. in Amami Oshima, there were four cases of emergency surgery and six cases of routine surgery among the cases of transfusion of donated blood in the hospital [8]. In our cases, when we analyzed the primary diagnosis that required emergency blood transfusion by classifying them according to the transfusion method, all cases of ABO-incompatible transfusion were emergencies, and the primary diagnosis of eleven out of twelve was gastrointestinal hemorrhage. Contrary to our concerns, none of the ABO-incompatible transfusions developed hemolytic anemia or acute kidney injury (AKI). On the other hand, the primary diagnosis of cases with inhospital-donated transfusion varied widely from gastrointestinal hemorrhage and postoperative bleeding to emergency obstetric bleeding. In addition, private hospitals do not have blood irradiation facilities for inhospital transfusions to prevent chronic graft-versus-host disease (GVHD). However, in the case of SI class II, inhospital donor transfusion has a high lifesaving rate and appears to be a necessary treatment option in remote islands with inadequate coagulation factors and/or platelet reserves (Table 7). In resource-limited settings, fresh whole blood transfusion from hospital donors may still be considered as an alternative [9].

### 5.4. Donor Profiles for Hospital-Donated Transfusion

For blood transfusion in our hospital, the donors were medical staff, family members, and neighbors, based on the goodwill of the islanders, and a comparable situation existed in Amami Oshima Island [10]. In Tokunoshima Tokushukai, gastrointestinal bleeding is the main cause of critical blood transfusions shown in the results; it may be necessary to promote endoscopic screening and health checkups by certified gastroenterologists.

### 5.5. Difficulty Determining Appropriate Storage Volume of Transfusion Products in Remote Island Healthcare Facilities

Despite the usefulness of modality, which relies solely on the goodwill of hibernators, forecasting and implementing adequate stockpiles of transfusion products will be an even greater challenge in the future. More than ever, remote islands are experiencing an aging population with a declining birth rate, and the population decline is relentless. With only a small number of people still living on remote islands, there is an urgent need to preserve medical resources and improve the local healthcare system. A certain amount of stockpiling is necessary on remote islands where blood transfusions are not available in an emergency. However, stock levels need to be adjusted to reduce wasteful stockpiling and make better use of finite medical resources. In addition, it is extremely difficult to determine the appropriate amount of blood transfusions to stockpile for unexpected bleeding outside of scheduled surgeries and emergencies, such as gastrointestinal bleeding or trauma. In our hospital, we have empirically stored two units of type A and B and six units of type O blood, and with permission for ABO-incompatible transfusions, in a medical environment without specialized full-time medical staff, such as gastroenterologists, surgeons, orthopedic surgeons, or transfusion medicine specialists. We hope to establish a unique and appropriate emergency medical system in the remote islands that will be well organized to promote the health of the islanders and continue to be the island of longevity.

### 5.6. Strength and Limitations

The strength of this study is that patients with hemorrhagic shock requiring urgent blood transfusion, as seen in the COVID-19 pandemic, should future pandemics and disasters disrupt the supply of transfusion products for a long period of time. We have been able to suggest the possibility of saving lives through inhospital transfusion and ABO-mismatched transfusion. We have also shown that they can have an economic impact on healthcare.

Next, we must mention the limitations of this study. First, due to the small number of studies, we could not prove the universal effectiveness of inhospital and ABO-incompatible transfusion methods. Second, the presence or absence of involvement of hemolytic anemia due to ABO-incompatible transfusion of 50 mL or more in the cause of death in the fatal cases was not fully verified [11]. However, both deaths are considered due to hemorrhagic shock rather than the onset of acute kidney injury due to hemolytic anemia. However, it cannot be excluded that hemolytic anemia was not the cause of death. Third, the method of inhospital donor transfusion shown to be effective in this study relies on the goodwill of donors, so this method of transfusion can be affected by a decrease in the number of donors due to population decline or a shortage of donors due to long-term pandemics and disasters. It is also necessary to consider the limitations of the continued use of this transfusion system in remote islands.

## 6. Conclusion

On remote islands, it is essential to store blood transfusions that can be used in emergencies, and by changing the stockpile of blood products in the hospital, the waste rate can be reduced. We changed the storage system of blood products for emergency transfusion in Tokunoshima in view of the withdrawal of the blood storage station in the Amami Islands. Comparing two consecutive 5-year periods before and after the intervention, which were set at the time of the withdrawal of the blood storage station in Amami, the reduction of blood product waste and the subsequent effectiveness of hospital-donated and ABO-incompatible transfusions as an alternative to emergency transfusion were demonstrated.

## Figures and Tables

**Table 1:** Blood transfusion standing volume preintervention and postintervention.

Periods	Blood types	Type A	Type B	Type O	Type AB
Preintervention 2015.1∼2017.12	Preintervention (unit)	4	4	4	2
Postintervention 2018.1∼2022.12	Postintervention (unit)	2	2	6	0

At Tokunoshima Tokushukai Hospital (TTH) since 2018, before (preintervention) and after (postintervention), type A and B decreased from four units to two units and type AB decreased from two units to zero units for transfusion products donated by the Japanese Red Cross Society. Conversely, type O for ABO-incompatible transfusion increased from four units to six units.

**Table 2:** Number of units transfused, volume discarded, discard rate, number of ABO-incompatible transfusions, and number of inhospital-donated transfusions from 2013 to 2023.

	Unit purchased	Amount disposed (USD)	Disposal rate (%)	ABO-incompatible transfusion	Inhospital-donated transfusion
*Preintervention*					
2013	910	11,202.10	20	0	2
2014	858	14,180.80	26.1	0	0
2015	932	14,687.20	24.9	0	1
2016	762	13,674.30	28.3	7	2
2017	706	7,976.70	17.8	0	0
Average	833.6	12,344.20	23.4		

*Postintervention*					
2018	792	7,850.00	15.7	4	0
2019	654	7,850.00	19	1	1
2020	716	7,641.30	16.5	1	0
2021	600	9,195.50	23.7	0	1
2022	670	6,346.20	14.6	4	2
Average	686.4	7,776.60	17.9		

The table represents the waste rate of donated blood products at JRCS in the preintervention and postintervention periods. As a result, the postintervention waste rate decreased from 23.4% to 17.9%, and the cost of wasted blood products was reduced by approximately 640,000 yen (4,571 USD) per year.

**Table 3:** The comparison of quantities and rates of disposal between two periods before and after the intervention^*∗*^.

Periods	2013∼2017 preintervention	2018∼2022 postintervention	*p* value
Unit purchased	858 (734, 921)	670 (627, 754)	0.047
Amount disposed (×100 USD)	136.74 (95.89, 144.34)	78.50 (69.94, 85.23)	0.016
Disposal rate (%)	24.9 (18.9, 27.2)	16.5 (15.2, 21.4)	0.047
Costs paid for transfused blood discarded (USD)	3674.30 (9589.40, 14434.00)	7850.00 (6993.75, 8522.75)	0.016

Comparing the results of ABO-incompatible transfusions before and after the intervention, the amount of purchased blood transfusions, the purchase cost of discarded blood transfusions, and the waste rate were all significantly reduced. ^*∗*^All data are expressed in the median (25% tile, 75% tile). Mann–Whitney's *U*-test was used for the nonparametric test of the null hypothesis.

**Table 4:** ABO-incompatible transfusion case profiles.

Years	Age	Gender	Disease	Treatment	Shock Index	Outcome	Blood type	Blood transfusion	Transfusion unit
*Preintervention period*									
2016	77	F	Bleeding duodenal ulcer	Endoscopic surgery	Class I	Survive	A^+^	O^+^	6
2016	39	M	Gastric varices rupture	Endoscopic surgery	Class II	Death	A^+^	O^+^	4
2016	63	M	Esophageal varices rupture	Endoscopic surgery	Class II	Death	B^−^	O^−^	6
2016	89	F	Neck fracture of thigh bone	Surgery	Class I	Survive	B^+^	O^+^	4
2016	75	M	Bleeding gastric ulcer	Endoscopic surgery, laparotomy	Class I	Survive	A^+^	O^+^	6
2016	70	F	Nonocclusive mesenteric ischemia	Experimental laparotomy	Class I	Death	A^+^	O^+^	2

*Postintervention period*									
2018	59	M	Bleeding gastric ulcer	Endoscopic surgery	Class II	Death	A^+^	O^+^	2
2018	84	M	Postoperative bleeding after gastric perforation	Endoscopic surgery	Class I	Survive	AB^+^	O^+^	2
2018	61	F	Fracture of the pelvis	Helicopter transport	Class I	Survive	A^+^	O^+^	2
2018	53	M	Esophageal varices rupture	Endoscopic surgery	Class II	Death	A^+^	O^+^	4
2020	91	M	Bleeding of gastric ulcer	Endoscopic surgery	Class I	Survive	A^+^	O^+^	2
2022	85	F	Bleeding from the digestive tract	Endoscopic surgery	Class I	Death	B^+^	O^+^	2

The number of cases of ABO-incompatible transfusion during the 5 years before and after the intervention was six each, and the mortality rate was 50% (3/6).

**Table 5:** Inhospital donor transfusion case profiles.

Years	Age	Gender	Disease	Treatment	Shock Index	Outcome	Blood type	Inhospital-donated transfusion	Inhospital transfusion unit
2013	73	M	Bleeding gastric ulcer	Endoscopic surgery	Class II	Survive	O^+^	O^+^	8
2013	28	F	Postpartum laxative hemorrhage	Emergency operation	Class II	Survive	AB^+^	AB^+^	4
2015	49	M	Esophageal varices rupture	Endoscopic surgery	Class III	Death	O^+^	O^+^	10
2016	82	F	Postoperative radius-ulnar fracture	Blood transfusion	Class I	Survive	A^+^	A^+^	2
2021	34	F	Early placenta abruption	Emergency operation	Class II	Survive (fatal death)	A^+^	A^+^	4

Case profiles of inhospital homozygous transfusions are presented. There were four cases before the intervention but only one case after the intervention. In other words, inhospital homozygous transfusion decreased after the intervention.

**Table 6:** Cases requiring ABO-incompatible transfusion and inhospital-donated blood transfusion.

Year	Age	Gender	Disease	Treatment	Shock Index	Outcome	Blood type	ABO-incompatible transfusion	unit	Inhospital blood transfusion (unit)
2016	83	F	Bleeding duodenal ulcer	Endoscopic surgery and laparotomy	Class I	Survive	A^+^	O^+^	2	2
2019	86	M	Bleeding after left artificial joint replacement surgery	Emergency operation	Class II	Survive	B^+^	O^+^	8	8
2022	48	M	Abdominal cattle trauma	Helicopter transport	Class II	Death	B^+^	O^+^	6	4
2022	70	M	Bleeding gastric ulcer	Endoscopic surgery	Class II	Death	A^+^	O^+^	6	18

Profiles of patients who underwent an inhospital homologous transfusion, followed by an ABO-incompatible transfusion, are presented. There was one case in the preintervention period, but this increased to three cases in the postintervention period, indicating that the number of cases of ABO-incompatible transfusion increased in the postintervention period. However, two of the three postintervention cases died. It may be necessary to re-evaluate the indications for blood transfusion in these fatal cases.

**Table 7:** Prognosis of Shock Index class II cases.

	Total	Survive	Death	Mortality rate (%)
Case of ABO-incompatible transfusion	8	1	7	87.5
Case of inhospital transfusion	5	4	1	20

The table shows the number of cases and mortality for ABO-incompatible and inhospital transfusions. Mortality was 87.5% with ABO-incompatible transfusion and 20% with inhospital transfusion. This suggests the effectiveness of inhospital transfusion.

## Data Availability

The data used to support the findings of this study are included within the article.
